# Supplementary material for: Molecular Docking and Dynamics Simulation Studies Predict Potential Anti-ADAR2 Inhibitors: Implications for the Treatment of Cancer, Neurological, Immunological and Infectious Diseases
Source: Int J Mol Sci. 2023 Apr 5;24(7):6795. doi: 10.3390/ijms24076795 (PMC10095294; doi:10.3390/ijms24076795)
Supplement: Supplementary file 1 [file ijms-24-06795-s001.zip › ijms-2252417-supplementary.pdf]

Supplementary Information

Molecular Docking and Dynamics Simulation Studies Predict Potential Anti-ADAR2 Inhibitors: Implications for the Treat-ment of Cancer, Neurological, Immunological and Infectious Diseases

Content

Figure S1: Plot of binding energy against molecular weight of the top 516 compounds (from the consensus scoring) via A) AutoDock Vina and B) Glide (Maestro).

Figure S2: Conformation and protein-ligand interaction profiles of the binding pose with the most negative binding energy of compounds A) ZINC000085950180, B) ZINC000085511995, C) ZINC000085850673, D) ZINC000085996580, E) ZINC000085734971, F) ZINC000014612330, G) ZINC000100513617 and H) ZINC000013462928. For the interaction profiles, purple arrows, red lines and combination of "red and blue" lines represent hydrogen bonds, pi-cation interactions and salt-bridges, respectively.

Figure S3: Root mean square deviation (RMSD), radius of gyration (Rg) and root mean square fluctuation (RMSF) plots of the unbound protein and ADAR2-ligand complexes after 100 ns MD simulations. (A) RMSD (B) Rg and (C) RMSF plot of the systems. The RMSF revealed less fluctuations in residues found in the active site region signifying strong and stable interactions between the ADAR2 and the ligands.

Figure S4: Chemical structures of the rest of the compounds in Table 2.

Table S1: Consensus docking scores and OSIRIS Datawarrior toxicity predictions of the 69 potential lead compounds with reasonably good pharmacokinetics profiles. Table cells with "None", "High" and "Low" classifications are highlighted green, red and yellow, respectively.

Table S2: Binding energies of the potential leads when docked against the 5-HT2CR via AutoDock Vina.

Supplementary Figures

A)

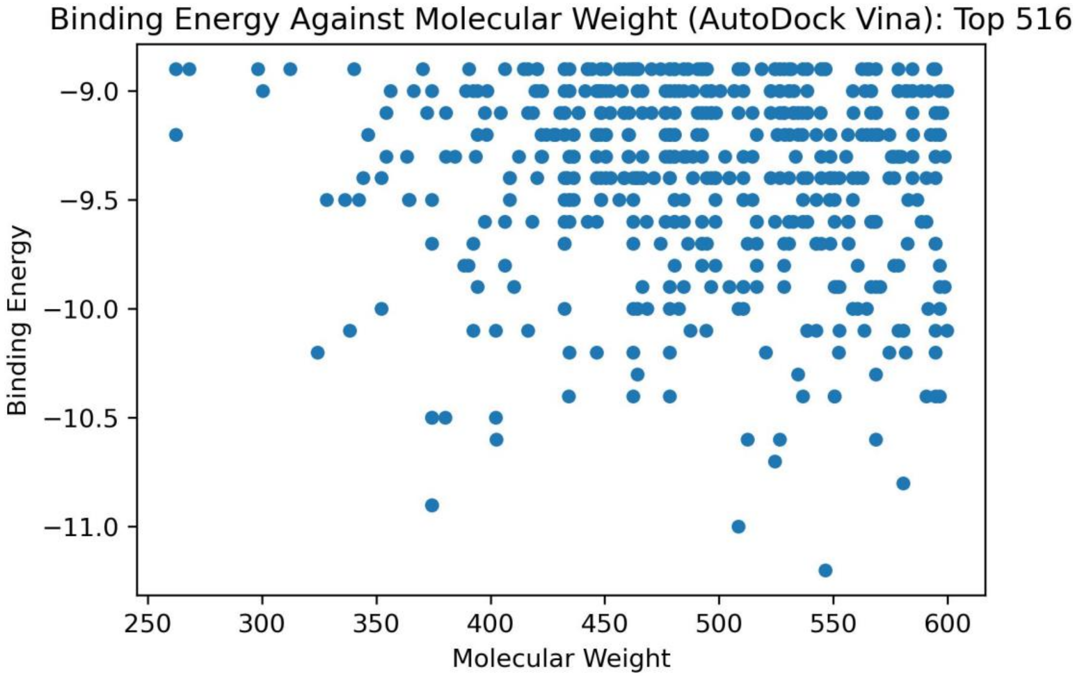

B)

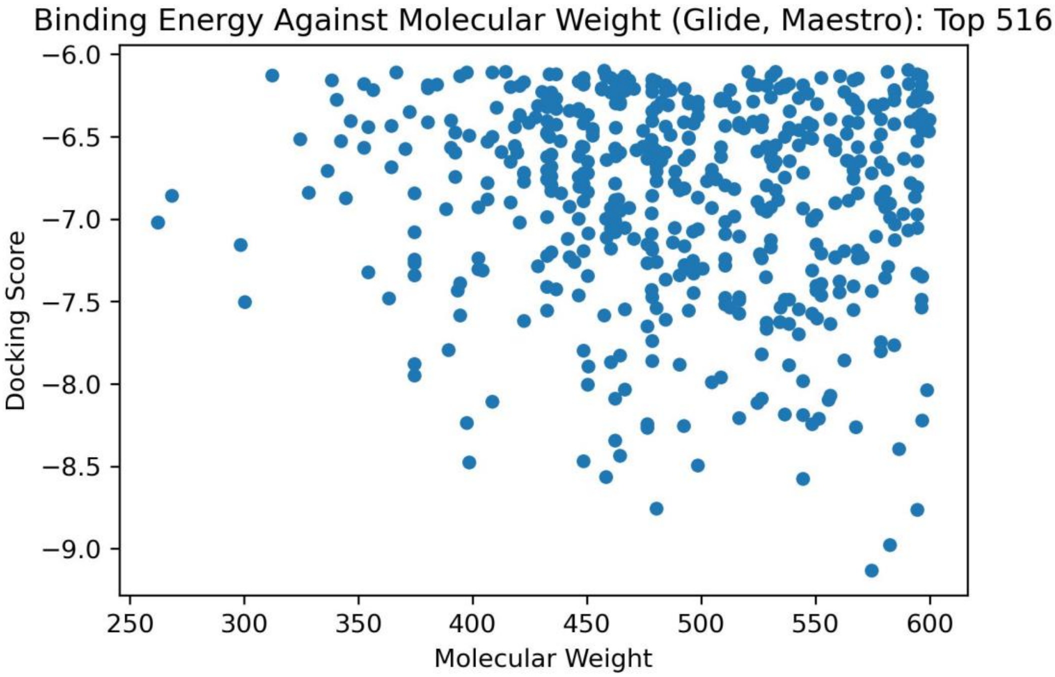

Figure S1: Plot of binding energy against molecular weight of the top 516 compounds (from the consensus scoring) via A) AutoDock Vina and B) Glide (Maestro).

A)

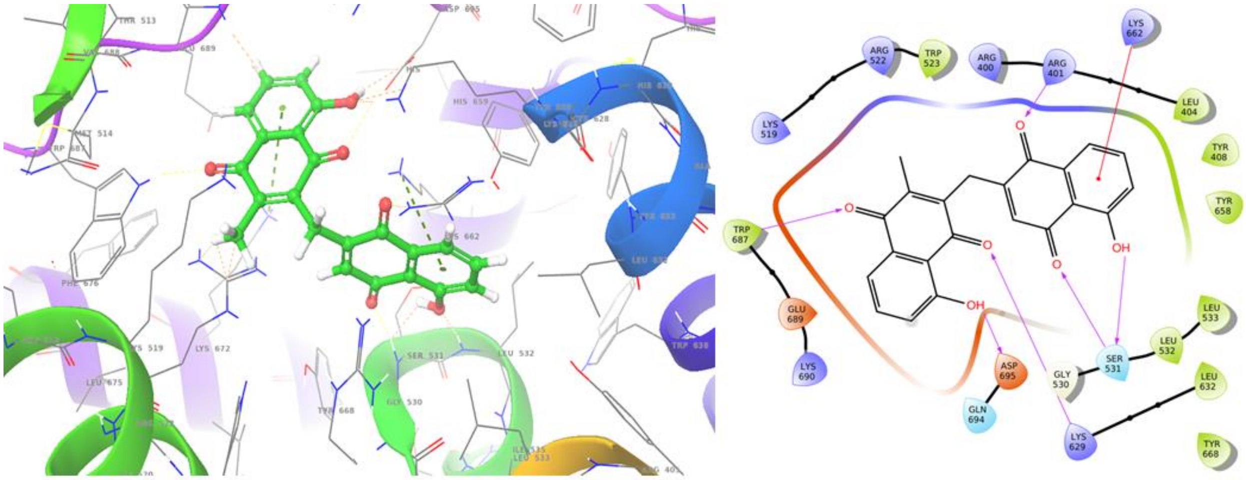

B)

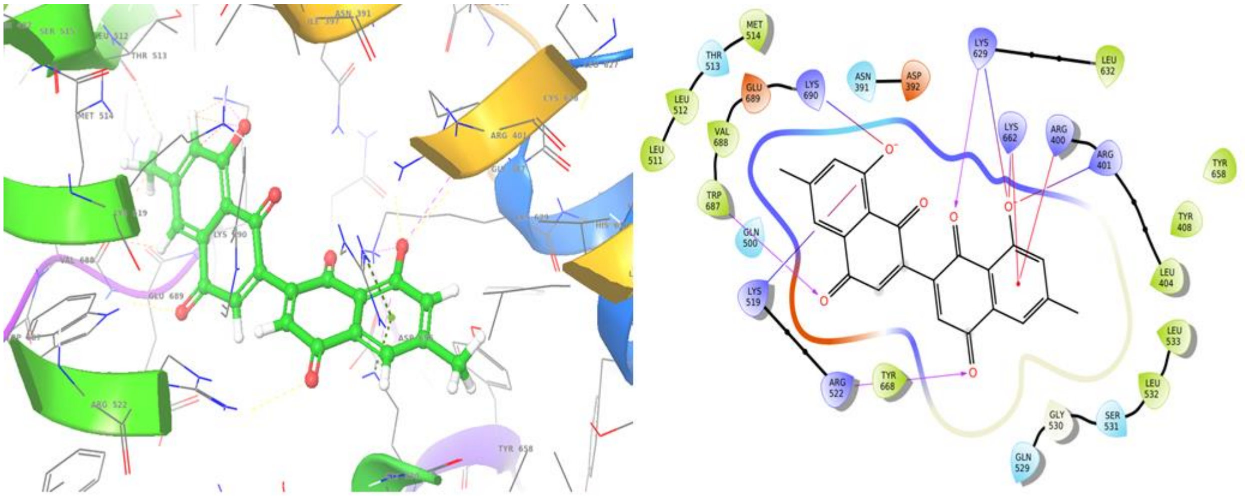
MET 514
THR 513
GLU 689
LYS 690
ASN 391
ASP 392
LYS 629
LEU 632
LEU 512
VAL 688
LEU 511
LYS 662
ARG 400
ARG 401
TYR 658
TRP 687
GLN 500
TYR 408
LYS 519
LEU 404
LEU 533
ARG 522
TYR 668
LEU 532
GLY 530
SER 531
GLN 529

C)

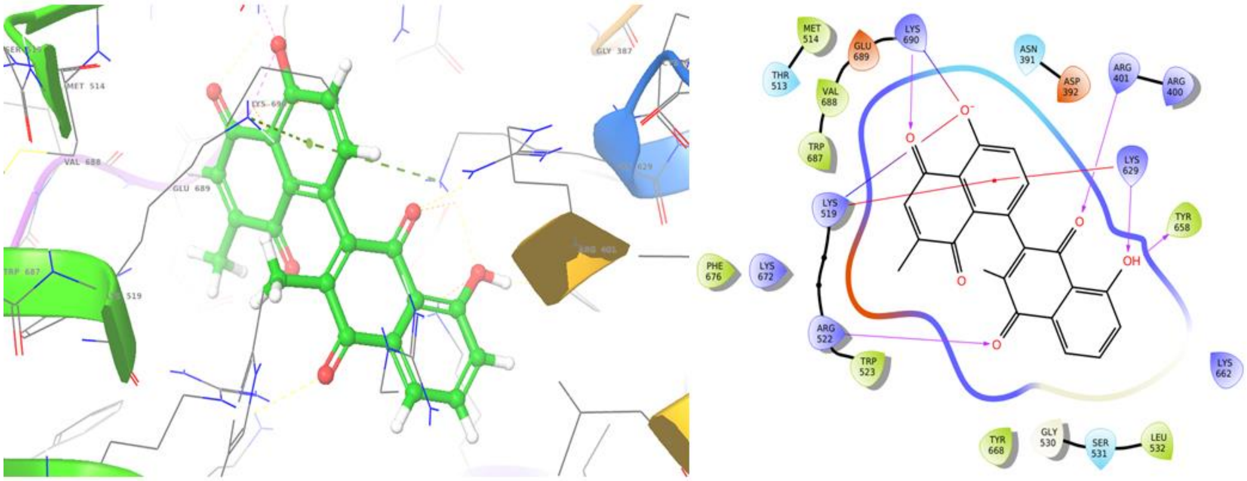
MET 514
GLU 689
LYS 690
THR 513
VAL 688
TRP 687
ASN 391
ASP 392
ARG 401
ARG 400
LYS 519
LYS 629
TYR 658
PHE 676
LYS 672
OH
ARG 522
TRP 523
LYS 662
TYR 668
GLY 530
SER 531
LEU 532

D)

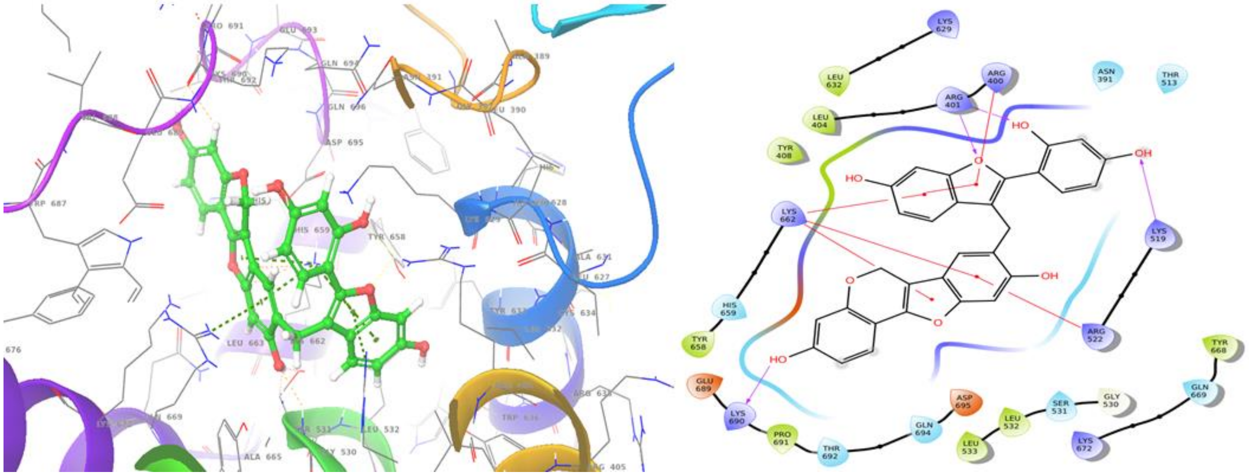
LYS 629
LEU 632
ARG 400
ARG 401
ASN 391
THR 513
LEU 404
HO
OH
TYR 408
HO
LYS 662
LYS 519
OH
HIS 659
ARG 522
TYR 658
HO
TYR 668
GLU 689
LYS 690
PRO 691
THR 692
GLN 694
ASP 695
LEU 533
LEU 532
SER 531
GLY 530
GLN 669
LYS 672

E)

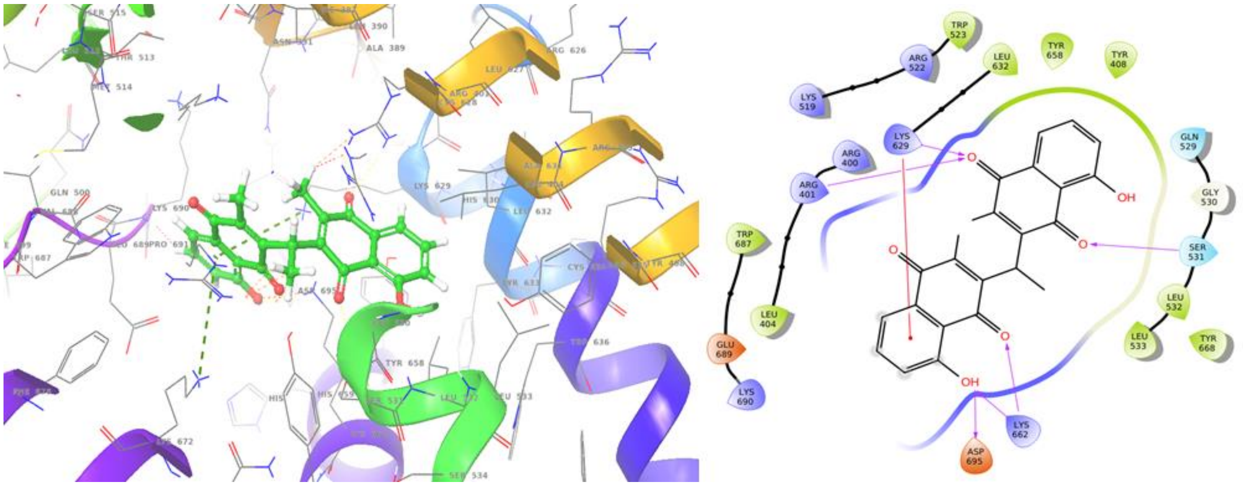
TRP 523
ARG 522
LEU 632
TYR 658
TYR 408
LYS 519
LYS 629
GLN 529
ARG 400
ARG 401
GLY 530
OH
SER 531
TRP 687
LEU 532
LEU 404
LEU 533
TYR 668
GLU 689
LYS 690
OH
LYS 662
ASP 695

F)

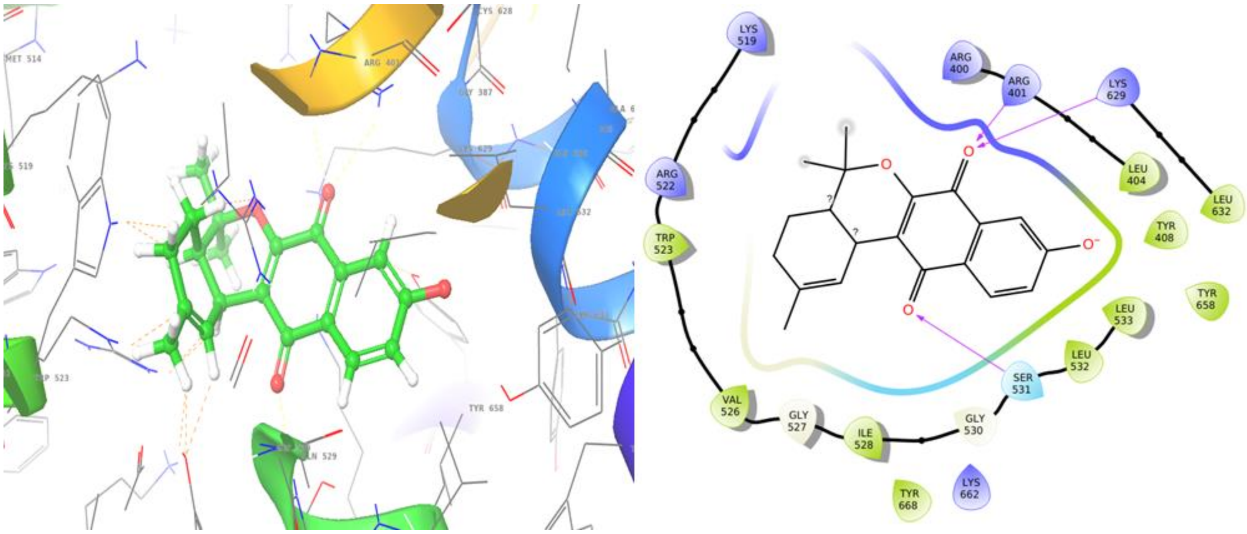
LYS 519
ARG 400
ARG 401
LYS 629
ARG 522
LEU 404
LEU 632
TRP 523
TYR 408
TYR 658
LEU 533
LEU 532
SER 531
VAL 526
GLY 527
ILE 528
GLY 530
TYR 668
LYS 662

G)

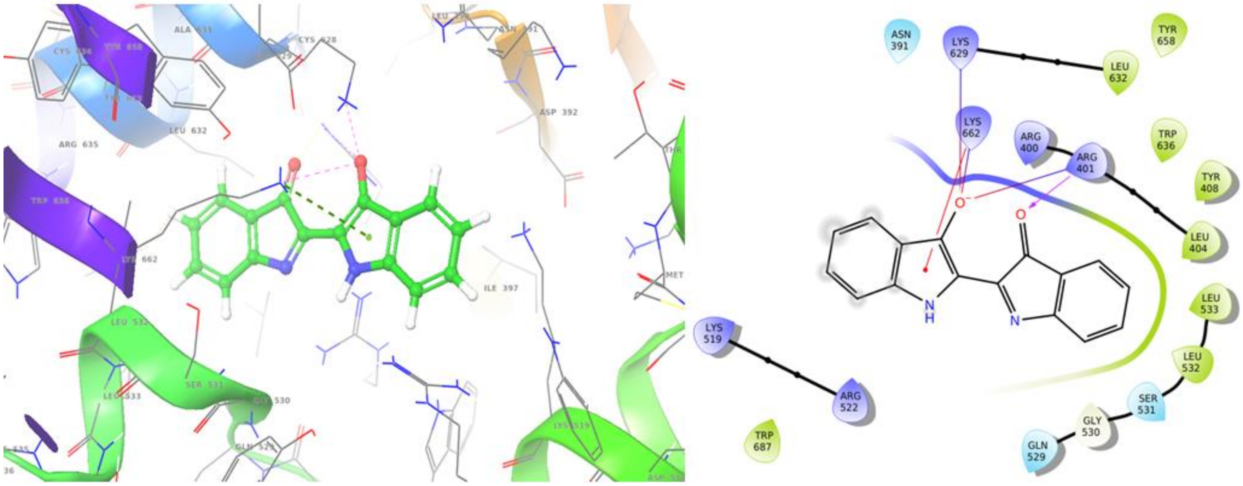
ASN 391
LYS 629
TYR 658
LEU 632
LYS 662
ARG 400
ARG 401
TRP 636
TYR 408
LEU 404
H
N
N
LEU 533
LYS 519
LEU 532
ARG 522
SER 531
TRP 687
GLY 530
GLN 529

H)

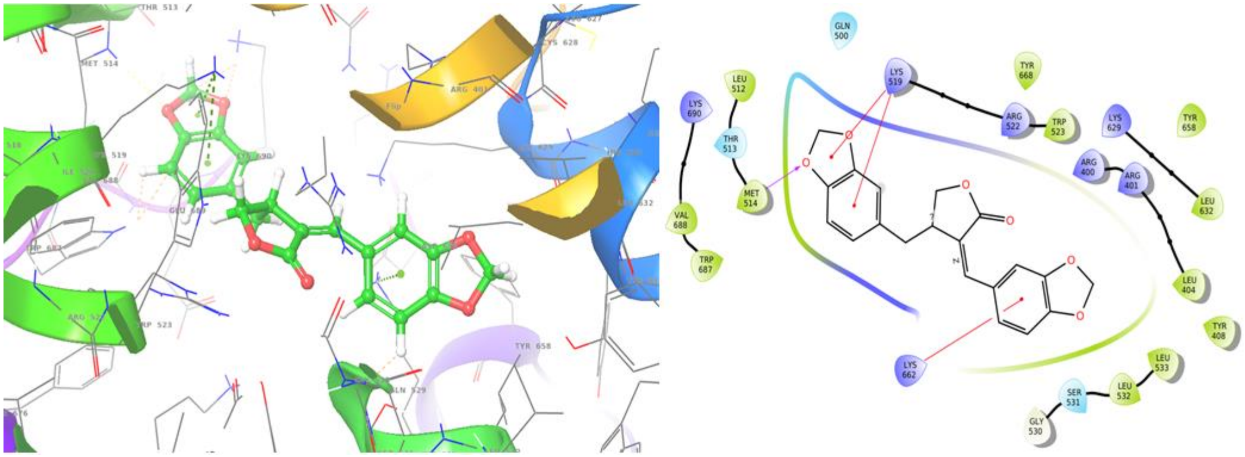

Figure S2: Conformation and protein-ligand interaction profiles of the binding pose with the most negative binding energy of compounds A) ZINC000085950180, B) ZINC000085511995, C) ZINC000085850673, D) ZINC000085996580, E) ZINC000085734971, F) ZINC000014612330, G) ZINC000100513617 and H) ZINC000013462928. For the interaction profiles, purple arrows, red lines and combination of "red and blue" lines represent hydrogen bonds, pi-cation interactions and salt-bridges, respectively.

A)

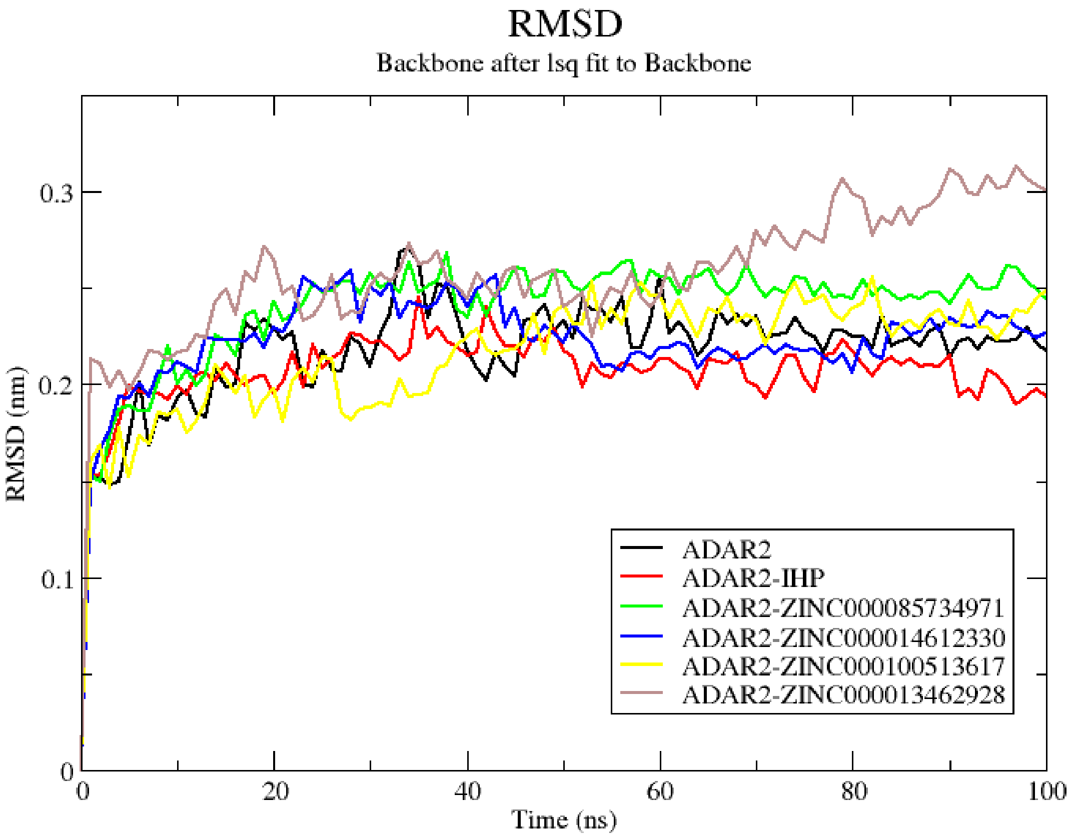
RMSD
Backbone after lsq fit to Backbone
RMSD (nm)
Time (ns)
0
0.1
0.2
0.3
0
20
40
60
80
100
ADAR2
ADAR2-IHP
ADAR2-ZINC000085734971
ADAR2-ZINC000014612330
ADAR2-ZINC000100513617
ADAR2-ZINC000013462928

B)

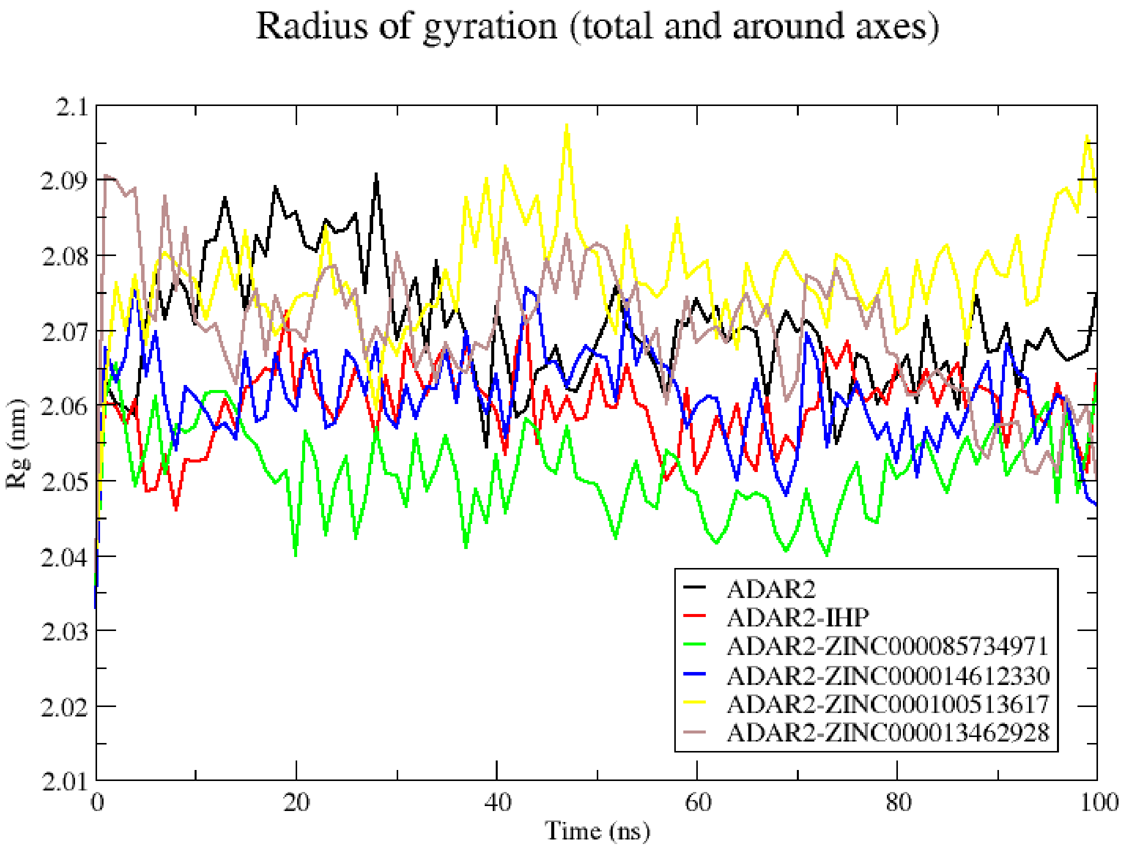
Radius of gyration (total and around axes)
2.1
2.09
2.08
2.07
2.06
2.05
2.04
2.03
2.02
2.01
Rg (nm)
0
20
40
60
80
100
Time (ns)
ADAR2
ADAR2-IHP
ADAR2-ZINC000085734971
ADAR2-ZINC000014612330
ADAR2-ZINC000100513617
ADAR2-ZINC000013462928

C)

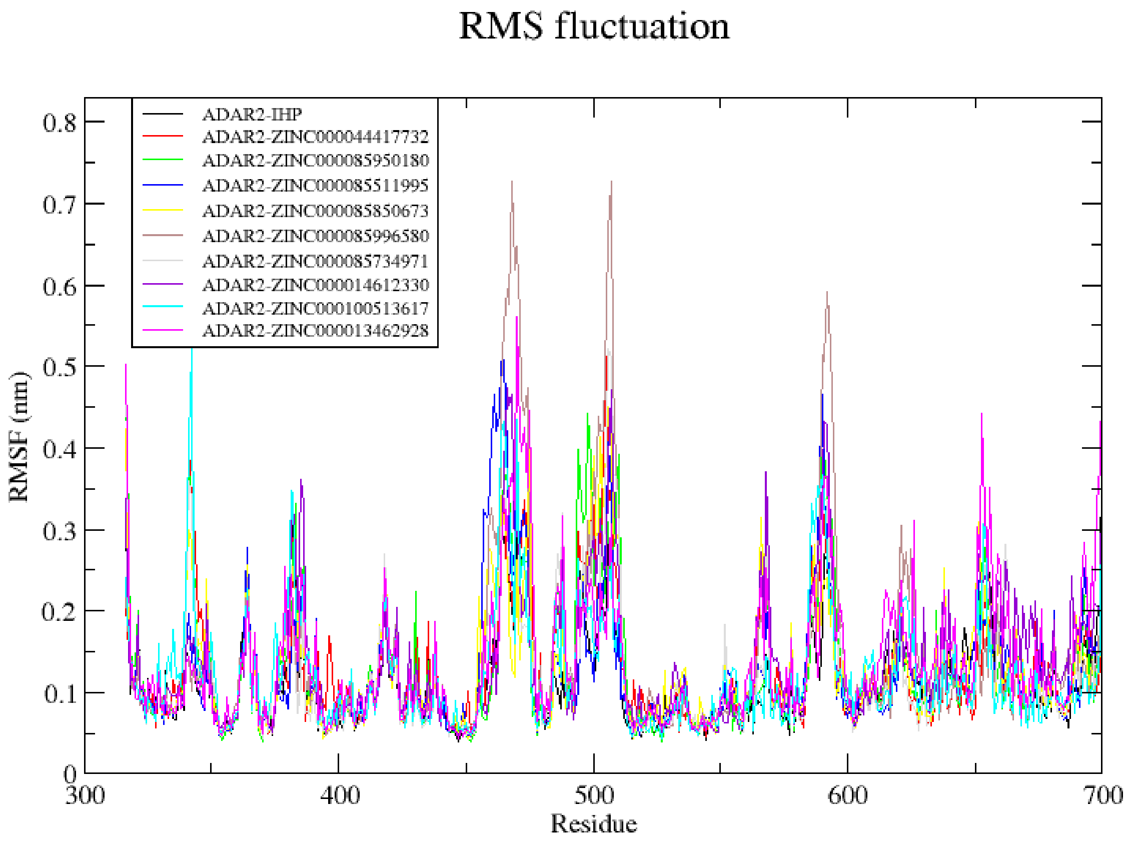

Figure S3: Root mean square deviation (RMSD), radius of gyration (Rg) and root mean square fluctuation (RMSF) plots of the unbound protein and ADAR2-ligand complexes after 100 ns MD simulations. (A) RMSD (B) Rg and (C) RMSF plot of the systems. The RMSF revealed less fluctuations in residues found in the active site region signifying strong and stable interactions between the ADAR2 and the ligands.

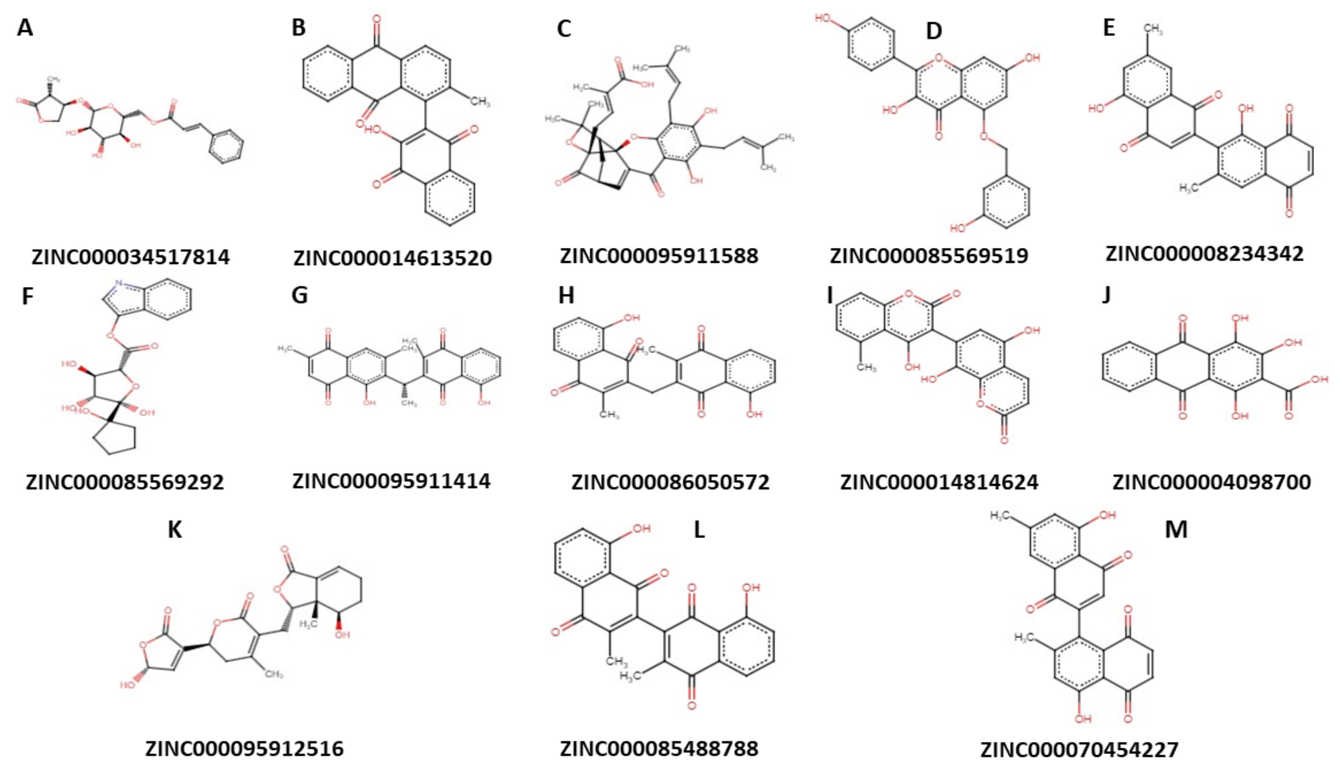

Figure S4: Chemical structures of the rest of the compounds in Table 2.

Supplementary Tables

Table S1: Consensus docking scores and OSIRIS Datawarrior toxicity predictions of the 69 potential lead compounds with reasonably good pharmacokinetics profiles. Table cells with "None", "High" and "Low" classifications are highlighted green, red and yellow, respectively.

Compound	Consensus Docking Score	Mutagenic	Tumorigenic	Irritant	Reproductive Effect
ZINC000044417732	-9.424132277	None	None	None	None
ZINC000085950180	-9.189206515	None	None	None	None
ZINC000085511995	-9.083019591	None	None	None	None
ZINC000085850673	-8.871610427	None	None	None	High
ZINC000085996580	-8.810739897	None	None	None	High
ZINC000085734971	-8.764515982	None	None	None	None
ZINC000034517814	-8.754400007	None	None	High	None
ZINC000014613520	-8.644636775	Low	None	High	None
ZINC000095911588	-8.628725069	None	None	None	High
ZINC000085569519	-8.421305685	High	None	None	None
ZINC000008234342	-8.389787435	None	None	None	None
ZINC000085569292	-8.389513991	None	None	None	None
ZINC000095911414	-8.37542394	None	None	None	None
ZINC000086050572	-8.368778188	None	None	None	None
ZINC000014612330	-8.356767518	None	None	None	None
ZINC000014814624	-8.283046901	Low	None	High	High
ZINC000004098700	-8.252200993	Low	None	High	None
ZINC000095912516	-8.183844059	Low	None	None	None
ZINC000085488788	-8.172253027	None	None	None	None
ZINC000070454227	-8.170951771	None	None	None	High
ZINC000013378578	-8.169429139	None	None	None	High
ZINC000015214955	-8.136074968	Low	None	None	None
ZINC000103579759	-8.12789818	None	None	None	None
ZINC000100513617	-8.109951671	None	None	None	None
ZINC000100017459	-8.1036925	None	None	None	None
ZINC000015254000	-8.093156447	Low	None	High	Low
ZINC000095911781	-8.054786976	None	None	None	None
ZINC000095911983	-8.027689262	Low	None	High	None
ZINC000013378580	-8.013173606	None	None	Low	High
ZINC000014454969	-8.000128717	None	None	High	None
ZINC000013379151	-7.970985095	None	None	None	Low
ZINC000014589300	-7.966771183	None	None	None	High
ZINC000085509705	-7.965240493	None	None	None	High
ZINC000095914424	-7.962915559	None	None	None	None
ZINC000100014196	-7.960751851	None	None	None	None
ZINC000085569270	-7.94949153	None	None	None	None
ZINC000085569501	-7.94661089	High	None	None	None
ZINC000003918875	-7.879107772	High	None	High	None

ZINC000014690579	-7.871547314	None	None	None	None
ZINC000085569417	-7.841426619	High	None	None	None
ZINC000004215683	-7.801833937	None	None	None	None
ZINC000085532383	-7.800595891	None	None	None	None
ZINC000085569484	-7.798554446	High	None	None	None
ZINC000085594490	-7.791704201	High	Low	None	None
ZINC000013462928	-7.790177528	None	None	None	High
ZINC000085541078	-7.780420675	None	None	High	None
ZINC000095912505	-7.779649507	None	None	None	None
ZINC000070454860	-7.759985149	None	None	None	None
ZINC000085645027	-7.759431157	None	None	None	None
ZINC000031597169	-7.754204662	None	None	None	None
ZINC000085508454	-7.753507879	None	None	High	None
ZINC000085569502	-7.746503785	High	None	None	None
ZINC000095914427	-7.741135858	None	None	High	None
ZINC000033832248	-7.736811479	None	None	None	None
ZINC000085569474	-7.73672787	High	None	None	None
ZINC000003979002	-7.723704809	None	None	None	High
ZINC000014721724	-7.695381938	None	None	High	None
ZINC000103543244	-7.666396706	None	Low	Low	None
ZINC000095909432	-7.655237256	None	None	None	None
ZINC000059586481	-7.651132524	None	None	None	None
ZINC000006095580	-7.642973788	None	None	High	None
ZINC000085970028	-7.620633781	None	None	None	High
ZINC000013485149	-7.608347346	None	None	None	None
ZINC000085486897	-7.604504173	None	None	High	High
ZINC000095911779	-7.571288517	None	None	None	None
ZINC000059588402	-7.557084706	High	High	None	None
ZINC000059587863	-7.554203147	Low	Low	High	None
ZINC000005714910	-7.513063127	Low	None	None	Low
ZINC000085569204	-7.50319783	None	High	None	High

Table S2: Binding energies of the potential leads when docked against the 5-HT2CR via AutoDock Vina.

**Compound**	**Name**	**Binding Energy**
Ritanserin	Ritanserin	-12.7
ZINC000044417732	Chitranone	-10.3
ZINC000085950180	Isozeylanone	-10.5
ZINC000085511995	Mamegakinone	-10.5

ZINC000085850673	3,8′-Bi[2-methyl-5-hydroxy-1,4-naphthoquinone]	-10.9
ZINC000085996580	8-[[2-(2,4-dihydroxyphenyl)-6-hydroxy-1-benzofuran-3-yl]methyl]-6H-[1]benzofuro[3,2-c]chromene-3,9-diol	-11.5
ZINC000085734971	3,3′-Ethylidenebis(2-methyl-5-hydroxy-1,4-naphthoquinone)	-10.6
ZINC000014612330	(4aR,12bR)-9-hydroxy-2,5,5-trimethyl-3,4,4a,12b-tetrahydronaphtho[3,2-c]isochromene-7,12-dione	-10.3
ZINC000100513617	Indigo	-9.6
ZINC000013462928	BDBM512896 or 3-(1,3-Benzodioxole-5-ylmethyl)-4,5-dihydrofuran-2(3H)-one	-9.9
